# Pathological features of BRCA-mutated breast cancer in Shenzhen, China: a single-center study

**DOI:** 10.7717/peerj.20813

**Published:** 2026-03-03

**Authors:** Jiayu Guan, Sihang Lin, Yanjia Liu, Wenbin Zhou

**Affiliations:** 1 The First Affiliated Hospital, Southern University of Science and Technology; The Second Clinical Medical College, Jinan University, Shenzhen People’s Hospital, Shenzhen, Guangdong, China; 2 Guangxi Medical University Cancer Hospital, Key Laboratory of Breast Cancer Diagnosis and Treatment Research of Guangxi Department of Education, Nanning, Guangxi, China

**Keywords:** Breast cancer, BRCA, NGS, Pathology, Molecular subtype

## Abstract

**Background:**

Most studies on breast cancer susceptibility gene (BRCA) mutations have focused on Caucasian populations in Europe and North America. Currently, there is a lack of literature and data research in related fields in Shenzhen, China, and even in Guangdong Province. This study aims to establish a registry of BRCA mutation carriers by analyzing and comparing the pathological features of breast cancer patients carrying and not carrying BRCA mutations in the Shenzhen area.

**Methods:**

Blood samples were collected from 406 breast cancer patients who met the inclusion criteria (from July 2016 to November 2024) and genetic testing was performed using next-generation sequencing (NGS) technology. Patients were divided into two groups: BRCA mutation group with 54 cases and BRCA non-mutation group with 352 cases. A retrospective analysis was conducted on patient data collected from the health information system of Shenzhen People’s Hospital, including demographic data, clinical pathological characteristics, and variables related to molecular typing. We used SPSS software for statistical analysis of the data.

**Results:**

In 406 breast cancer patients, the average age of the BRCA mutation group was 39.3 ± 9.2 years, while the average age of the BRCA non-mutation group was 41.8 ± 8.8 years. The proportion of tumors ≤ 2 cm in the mutation group is 72.2%, higher than the 53.1% in the non-mutation group (*P* = 0.009, 95% confidence interval [1.220–4.313]). The proportion of grade III pathologic grading in the mutation group is 59.3%, higher than the 36.1% in the non-mutation group (*P* = 0.001, 95% confidence interval [1.436–4.625]). In the mutation group, there are seven cases of Luminal A (13.0%), zero cases of Luminal B (Her-2 positive) (0%), and 23 cases of triple-negative breast cancer (TNBC) (42.6%). In the non-mutation group, there are 93 cases of Luminal A (26.4%), 54 cases of Luminal B (Her-2 positive) (15.3%), and 67 cases of TNBC (19.0%). (Luminal A: *P* = 0.033, 95% confidence interval [0.181–0.950]; Luminal B (Her-2 positive): *P* = 0.002; TNBC: *P* < 0.001, 95% confidence interval [1.730–5.759]). The expression levels of estrogen receptor (ER) (*P* = 0.009), progesterone receptor (PR) (*P* < 0.001), and Ki-67 (*P* < 0.001) show significant differences between the BRCA mutation group and the BRCA non-mutation group.

**Conclusions:**

Compared to BRCA non-mutated patients, BRCA mutated patients in Shenzhen have smaller tumor volumes, with pathological grades mainly at grade 3, and molecular subtypes predominantly being triple-negative breast cancer.

## Introduction

Breast cancer is the most common malignant tumor among women worldwide, with over 2 million new cases diagnosed each year, and it is a leading cause of cancer-related deaths in women (Sung et al., 2021). The occurrence of breast cancer is influenced by various factors, including hormone levels, genetics, lifestyle, and environmental factors (Rojas & Stuckey, 2016). Estrogens are considered significant risk factors for breast cancer, as they can promote the proliferation of breast cells (Nuñez-Olvera et al., 2022). Breast cancer susceptibility gene (BRCA) mutations are regarded as important genetic factors that significantly increase the risk of breast cancer (Lerner-Ellis et al., 2021). As BRCA genes play a crucial role in DNA damage repair, particularly in the homologous recombination repair pathway (Arun et al., 2024). Patients carrying BRCA1 or BRCA2 mutations have an approximate breast cancer risk of 78% and 56%, respectively (Agostinetto et al., 2024). At the same time, the risk of developing ovarian cancer is also significantly increased.

In clinical practice, histological grading, lymph node status, Ki-67 proliferation index, and molecular typing are all considered important indicators affecting prognosis (Zhou et al., 2024). Research shows that patients with BRCA mutations typically have a higher histological grade of tumors, and the incidence of lymph node metastasis is higher among these patients. In addition, the Ki-67 proliferation index of breast cancer in BRCA mutation patients is usually higher (Kim, Choi & Park, 2020). Breast cancer associated with BRCA1 mutations typically occurs at a younger age and is mainly triple-negative breast cancer, which has a poor clinical prognosis (Telli et al., 2024). In contrast, breast cancer associated with BRCA2 mutations is more likely to be hormone receptor-positive, with a relatively better prognosis (Tutt et al., 2021). Increasing evidence suggests that the prognosis of BRCA mutation carriers is worse than that of non-carriers (Bao et al., 2024; Vasigh et al., 2022).

Although many studies have explored the impact of BRCA gene mutations on breast cancer, there is a lack of systematic analysis of the pathological features of breast cancer patients carrying BRCA gene mutations in specific areas such as Shenzhen and Guangdong Province. This limits the comprehensive assessment of the clinical significance of BRCA gene testing in high-risk women with breast cancer, including diagnostic value, treatment guidance, and prognostic evaluation (Bao et al., 2024). Therefore, a study on BRCA gene testing for high-risk women with breast cancer has been conducted in the Shenzhen area to guide the prevention and management strategies for breast cancer in this region. The results of this study will provide important evidence for improving the management and clinical decision-making for breast cancer patients in this area.

## Materials and Methods

Portions of this text were previously published as part of a preprint (Guan, Liu & Zhou, 2025).

### Study subjects

**Figure 1:**
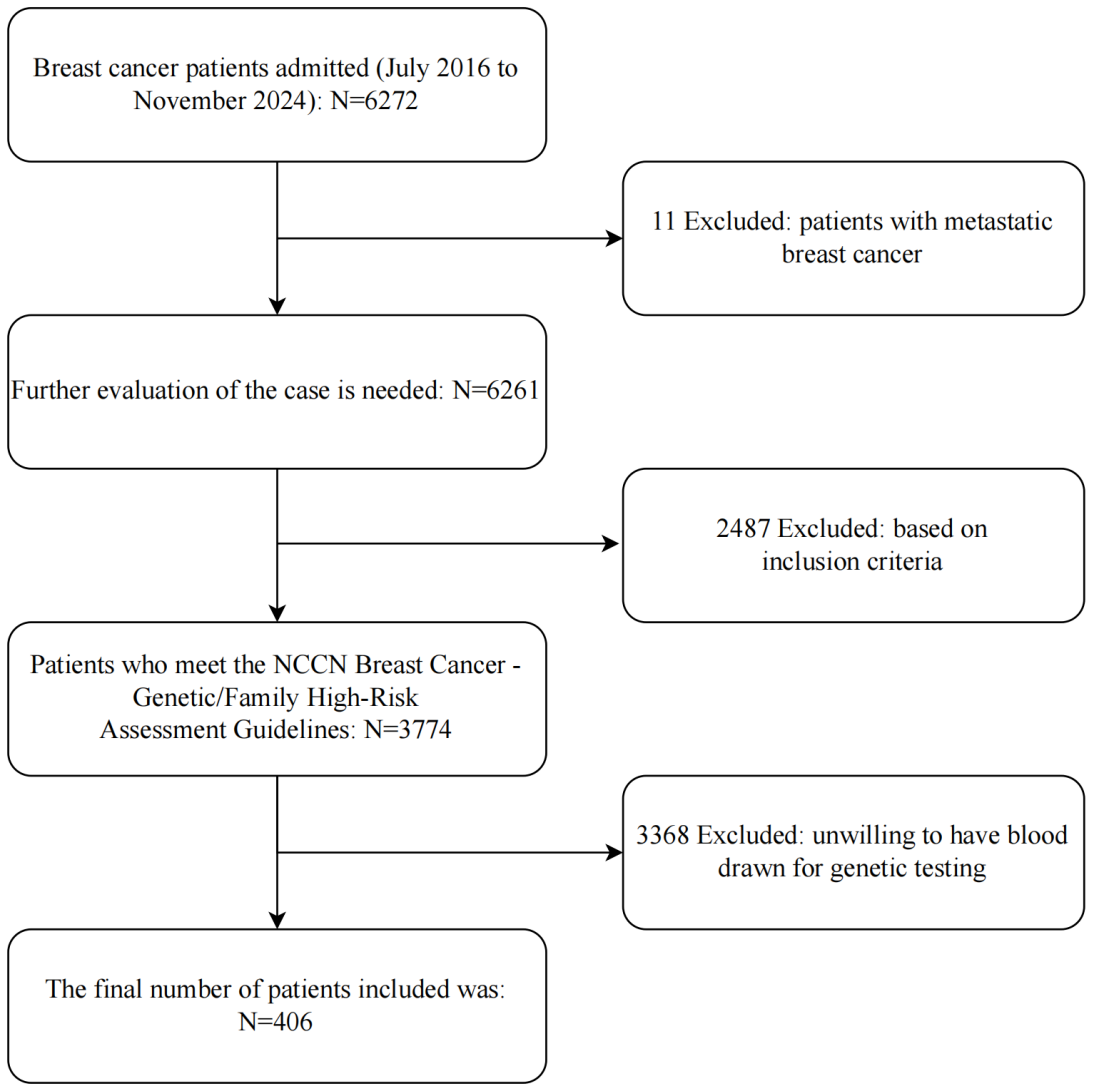
Flowchart of patient inclusion and exclusion.

This study included patients diagnosed with breast cancer in the Department of Breast Surgery at Shenzhen People’s Hospital from July 2016 to November 2024, collecting clinical and pathological data from a total of 406 eligible patients. The study was approved by the hospital’s Ethics Committee, ethical approval number: LL-KY-2025124-02, and written informed consent was obtained from the participants. Inclusion criteria: Comply with the standards for genetic and familial high-risk assessment of breast cancer and ovarian cancer in the National Comprehensive Cancer Network (NCCN) guidelines (Daly et al., 2021). Exclusion criteria: Patients with a pathological diagnosis confirmed as metastatic breast malignancy not originating from the mammary glands; patients unwilling to undergo blood tests for genetic testing. The inclusion and exclusion process of patients is shown in Fig. 1.

### BRCA gene detection methods

**Figure 2:**
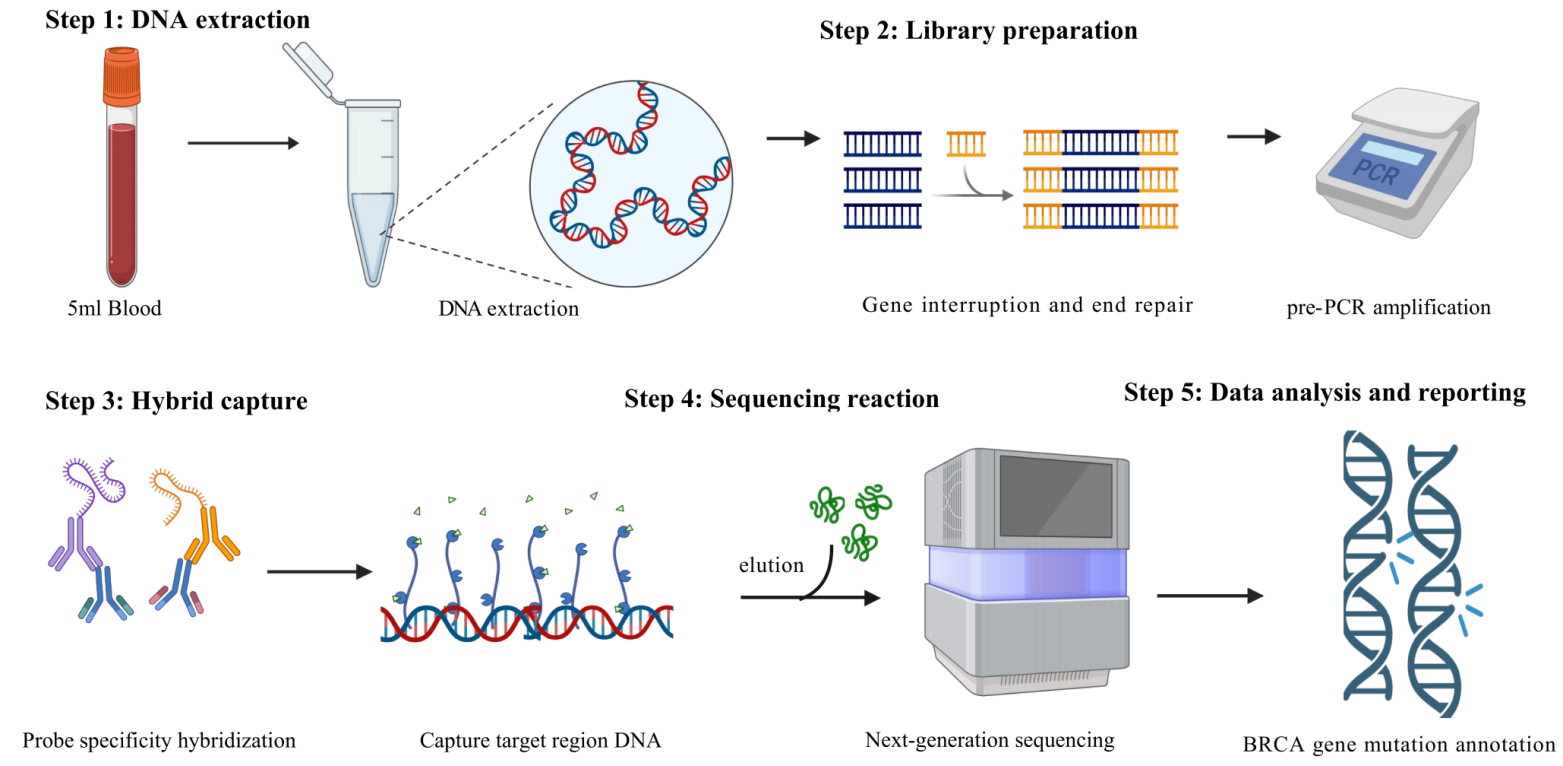
The gene testing process.

Five mL of peripheral blood sample in EDTA tube, genomic DNA was extracted using hereditary tumor gene detection kit (BGI Genomics, Shenzhen, China). The genomic DNA was fragmented and end-repaired through enzymatic reactions; adapters containing tag sequences were added to both ends of the DNA using ligase, and the pre-PCR library was formed through PCR amplification. The target DNA fragments in the library were hybridized with hereditary tumor probes containing the BRCA gene labeled with biotin. The target DNA fragments were anchored on streptavidin magnetic beads through biotin affinity reactions. The elution buffer (Roche, Basel, Switzerland) was used to remove non-target DNA. Specific capture of enriched DNA; the post-hybridization library was obtained through PCR amplification. The post-PCR library underwent single-strand separation, circularization, and rolling circle amplification to generate DNA nanoballs. Sequencing was performed using a gene sequencer, with the sequencing platform being MGISEQ2000. The data from the sequencer was filtered, aligned, deduplicated, and quality controlled. Variant analysis was conducted to obtain the variant analysis results (VCF file); the variant results were annotated using the BGI-HALOS integrated machine and core database to derive clinical grade classification (Fig. 2). The specific parameters of next-generation sequencing (NGS) can be seen in Table 1.

**Table 1:** The description of the next-generation sequencing (NGS) methodology.

Key performance indicators	Specific parameters
Chip size	1.1Mb
Sequencing depth	>150X
Detection content	SNP, InDel, CNV
Sequencing platform	MGISEQ2000 (European Union:DNBSEQ-G400)
Interpretation platform	BGI-HALOS integrated machine, Core database
Report content	Classification of test results and clinical significance (based on ACMG interpretation guidelines for 5 categories)
Sample requirements	EDTA peripheral blood 5 mL
Gene	BRCA1, BRCA2, CHEK2, PALB2, BRIP1, TP53, PTEN, STK11, CDH1, ATM, BARD1, MLH1, MRE11A, MSH2, MSH6, MUTYH, NBN, PMS1, PMS2, RAD50, RAD51C, RAD51D, NF1, EPCAM, SMARCA4, CDK12

### Immunohistochemical scoring criteria

The following standards refer to the 2021 Chinese Anti-Cancer Association Guidelines and Norms for Breast Cancer Diagnosis and Treatment (Chinese Anti-Cancer Association CoBCS, 2021). Estrogen receptor (ER) positive is defined as the proportion of estrogen receptor positive staining in tumor cells ≥1%; progesterone receptor (PR) positive is defined as the proportion of progesterone receptor positive staining in tumor cells ≥1%. Her-2 (3+) is defined as positive; Her-2 (1+) or Her-2 (0) is defined as negative. When Her-2 (2+) is present, further fluorescence *in situ* hybridization (FISH) testing is required. If the test results show Her-2 gene amplification, it is considered positive; otherwise, it is considered negative. A Ki-67 proliferation index ≥20% is defined as high expression level, while <20% is defined as low expression level. The five molecular subtypes of breast cancer include: Luminal A, Luminal B (Her-2 Positive), Luminal B (Her-2 Negative), Her-2 overexpression, and triple-negative breast cancer (TNBC), with specific classifications as shown in Table 2.

**Table 2:** Breast cancer molecular typing.

Type	ER (%)	PR (%)	Her-2	Ki-67 (%)
Luminal A	1–100	20–100	–	0–19
Luminal B (Her-2 Negative)	1–100	0–19	–	20–100
Luminal B (Her-2 Positive)	1–100	0–100	+	0–100
Her-2 overexpression	0	0	+	0–100
TNBC	0	0	–	0–100

**Notes.**

Certain hormone receptor-positive tumors that do not meet Luminal A criteria (such as ER-negative and PR-positive) can be considered Luminal B; Luminal B (Her-2 Negative): as long as either the progesterone receptor (PR) or the proliferation index (Ki-67) meets the criteria, the diagnostic conditions can be satisfied; “+”, Positive; “–”, Negative; TNBC, Triple negative breast cancer.

### Grouping

**Figure 3:**
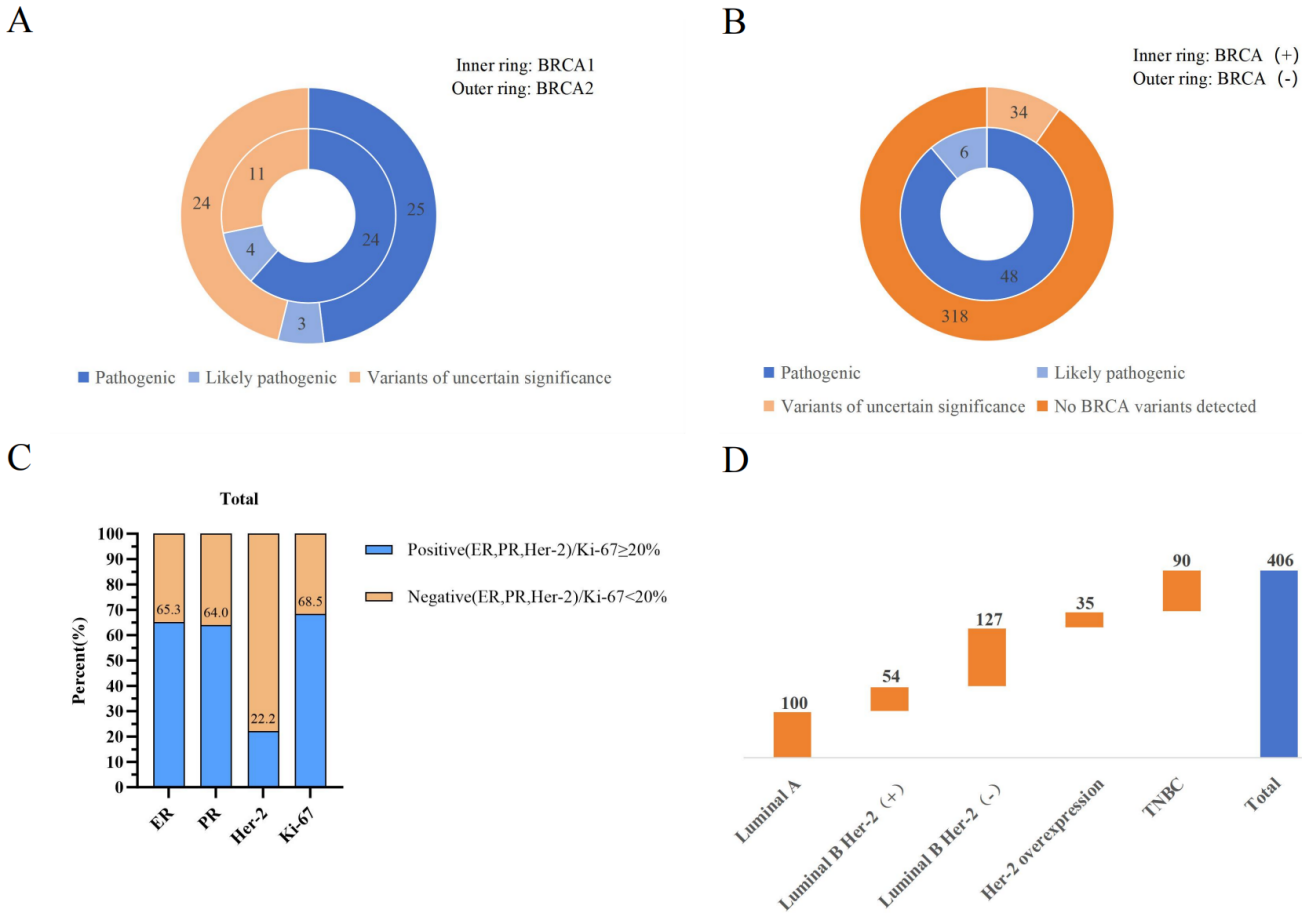
(A) Distribution of BRCA1 and BRCA2 gene mutation types; (B) the number of cases of BRCA(+) and BRCA(−) mutation types; (C) analysis of the positive and negative proportions of ER, PR, Her-2, and Ki-67 in enrolled patients; (D) analysis of molecular typing distribution in enrolled patients.

The BRCA mutation group includes patients carrying pathogenic or likely pathogenic variants (P/LPVs) of BRCA, that is, BRCA (+); the non-BRCA mutation group includes patients carrying variants of uncertain significance (VUS) and patients without detected BRCA mutations, that is, BRCA (-) (Fig. 3A).

### Statistical analysis

This study used SPSS version 27.0 for statistical analysis. Measurement data with a normal distribution are expressed as mean  ± standard deviation ($\bar {x}~\pm ~$s), and inter-group comparisons are performed using independent samples *t*-test. For measurement data with a non-normal distribution, the Mann–Whitney U test is used to compare differences between the two groups. Count data are expressed as rates or composition ratios, and inter-group comparisons are performed using Pearson’s chi-square test or Fisher’s exact test; a two-sided *P* value less than 0.05 is considered statistically significant.

## Results

### Basic information

A total of 406 breast cancer patients were included in this study, among which 54 were in the BRCA mutation group, including 53 females (98.1%) and one male (1.9%). The average age of this group of patients was 39.3  ± 9.2 years. There were 39 patients (72.2%) with tumor size ≤2 cm, and 50 patients (92.6%) with tumor stages ranging from stage 0 to stage II; there were 20 patients (37.0%) with positive lymph nodes. There were four patients (7.4%) with carcinoma *in situ*; 50 patients (92.6%) had invasive breast cancer. In histological grading, there were 17 patients (31.5%) in grade I–II and 32 patients (59.3%) in grade III. In the non-BRCA mutation group, there were a total of 352 patients, including 350 females (99.4%) and two males (0.6%). The average age of this group of patients was 41.8 ± 8.8 years. There were 187 patients (53.1%) with tumor size ≤2 cm; 312 patients (88.6%) had tumor stages ranging from Stage 0 to Stage II. There were 100 patients (28.4%) with positive lymph nodes. There were 46 patients (13.1%) with carcinoma *in situ*; 302 patients (85.8%) had invasive breast cancer. In histological grading, there were 209 patients (59.4%) in grade I–II and 127 patients (36.1%) in grade III (see Table 3). For detailed clinical and pathological data on these patients, please refer to Table S1.

**Table 3:** Comparison of clinical and pathological characteristics between the two groups (n(%)).

Characteristics	BRCA (+) *N* = 54	BRCA (-) *N* = 352	*P*-value	OR (95% CI)
Sex			0.349	3.302 (0.294–37.050)
Male	1 (1.9)	2 (0.6%)		
Female	53 (98.1)	350 (99.4%)		
Age	39.3 ± 9.2	41.8 ± 8.8	0.056	(−5.011–0.064)
Tumor size stage				
T1	39 (72.2)	187 (53.1)	0.009	2.294 (1.220–4.313)
T2	15 (27.8)	150 (42.6)	0.039	0.518 (0.275–0.974)
T3-T4	0 (0.0)	15 (4.3)	0.237	NA
TNM stage				
Stage 0	4 (7.4)	43 (12.2)	0.304	0.575 (0.198–1.671)
Stage I	25 (46.3)	126 (35.8)	0.137	1.546 (0.868–2.755)
Stage II	21 (38.9)	143 (40.6)	0.809	0.930 (0.517–1.673)
Stage III	4 (7.4)	37 (10.5)	0.481	0.681 (0.233–1.993)
Stage IV	0 (0.0)	3 (0.9)	1.000	NA
Lymph node metastasis			0.196	0.675 (0.371–1.228)
No	34 (63.0)	252 (71.6)		
Yes	20 (37.0)	100 (28.4)		
Pathological type				
DCIS	4 (7.4)	44 (12.5)	0.280	0.560 (0.193–1.627)
LCIS	0 (0.0)	2 (0.6)	1.000	NA
IDC	49 (90.7)	283 (80.4)	0.067	2.389 (0.918–6.222)
ILC	0 (0.0)	13 (3.7)	0.232	NA
IC-ST	1 (1.9)	6 (1.7)	1.000	1.079 (0.127–9.137)
Other	0 (0.0)	4 (1.1)	1.000	NA
Histological grading				
I	4 (7.4)	32 (9.1)	0.882	0.800 (0.271–2.359)
II	13 (24.1)	177 (50.3)	<0.001	0.313 (0.162–0.605)
III	32 (59.3)	127 (36.1)	0.001	2.577 (1.436–4.625)
other	5 (9.2)	16 (4.5)	0.260	2.143 (0.751–6.111)

**Notes.**

DCIS Ductal Carcinoma *In Situ* LCIS Lobular Carcinoma *In Situ* IDC Invasive Ductal Carcinoma ILC Invasive Lobular Carcinoma IC-ST Invasive Carcinoma of Specialtype NA Not applicable BRCA (+) BRCA mutation group BRCA (-) non-BRCA mutation group OR Odds ratio CI Confidence interval

### BRCA gene mutation types and molecular typing

Among the 54 patients with detected BRCA gene mutations, 48 cases (88.9%) had pathogenic variants, and six cases (11.1%) had likely pathogenic variants. In contrast, among 352 cases of non-BRCA mutation patients, 34 cases (9.7%) were variants of uncertain significance, while 318 cases (90.3%) showed no BRCA gene mutations. Two patients were found to carry mutations in both BRCA1 and BRCA2 genes. Specifically, pathogenic variants of BRCA1/2 were detected in 24 and 25 cases, variants of uncertain significance were detected in four and three cases, and variants of uncertain significance were detected in 11 and 24 cases. Among 406 patients, the proportions of ER-positive, PR-positive, Her-2 positive, and high Ki-67 expression were 65.3%, 64.0%, 22.2%, and 68.5%, respectively; there were 100 patients in the Luminal A group; 54 patients in the Luminal B (Her-2 positive) group; 127 patients in the Luminal B (Her-2 negative) group; 35 patients in the Her-2 overexpression group; and 90 patients in the TNBC group (Fig. 3).

### Comparison of clinical pathological features

In the BRCA mutation group, tumor size tended to be ≤2 cm, while in the non-mutation group, it tended to be 2–5 cm, with statistical significance (*P* = 0.009, *P* = 0.039), 95% confidence interval (1.220–4.313, 0.275–0.974). The histological grade of tumors in the BRCA mutation group tended to be grade III, while in the non-mutation group, it tended to be grade II, with statistically significant differences (*P* = 0.001, *P* < 0.001), 95% confidence interval (1.436–4.625, 0.162–0.605). No significant statistical significance was observed for other indicators (Table 3).

### Comparison of molecular subtypes of tumors

In the breast cancer group with BRCA mutations, there are seven cases of Luminal A (13.0%), and zero cases of Luminal B (Her-2 positive); there is one case of Her-2 overexpression (1.9%), and the number of cases for TNBC subtype and Luminal B (Her-2 negative) is the same, both are 23 cases (42.6%). In the breast cancer group without BRCA mutations, the number of cases for Luminal B (Her-2 negative) is the highest, with 104 cases (29.5%), Luminal A has 93 cases (26.4%), and Luminal B (Her-2 positive) has 54 cases (15.3%); there are 34 cases of Her-2 overexpression (9.7%), and 67 cases of TNBC subtype (19.0%). The distribution differences of Luminal A (*P* = 0.033), Luminal B (Her-2 positive) (*P* = 0.002), and TNBC subtype (*P* < 0.001) between the two groups are statistically significant (Table 4).

### Comparison of immunohistochemical markers

The comparison of ER, PR, and Ki-67 expression levels between the BRCA mutation group and the non-mutation group is detailed in Table 5. The Mann–Whitney U test analysis results indicate that there are significant differences between the two groups in ER, PR, and Ki-67 (*p* = 0.009, *p* < 0.001, and *p* < 0.001), specifically manifested as statistical differences in the expression levels of each indicator. It is worth noting that both groups showed statistically significant differences in the negative rates of ER, PR, and Her-2 (*p* = 0.032, 0.014, <0.001) as well as in the high expression of Ki-67 (*p* = 0.012). In summary, compared with non-BRCA mutant breast cancer, BRCA mutant breast cancer typically exhibits molecular characteristics of ER negative, PR negative, Her-2 negative, and high expression of Ki-67.

## Discussion

BRCA mutations are divided into germline mutations and somatic mutations (Vlessis et al., 2020). This study determined the BRCA mutation status by testing blood samples, and the results were all germline mutations. Germline mutations and somatic mutations differ in terms of incidence (Jin et al., 2022), age at onset (Chung et al., 2020; Winter et al., 2016), and chemotherapy response (Azaïs et al., 2024; Fasching et al., 2024), but show similar responses to PARP inhibitors treatment (Walsh et al., 2022) and tumor phenotypes (Winter et al., 2016). For example, the average age of onset for patients with germline BRCA1 and BRCA2 mutations is 41.5 years and 49.5 years, respectively, while the average age of onset for patients with somatic mutations is 65 years (Winter et al., 2016). The average age of BRCA mutation breast cancer patients in this study is below 40 years, which is generally consistent with the age characteristics of germline BRCA mutations. For a detailed comparison between germline and somatic mutation patients, see Table S2.

**Table 4:** Comparison of molecular subtypes of tumors between the two groups (n(%)).

Variable	BRCA(+) *N* = 54		BRCA(-) *N* = 352		*P*-value	OR (95% CI)
	Yes	No	Yes	No		
LuminalA	7 (13.0%)	47 (87.0%)	93 (26.4%)	259 (73.6%)	0.033	0.415 (0.181–0.950)
Luminal B Her-2 Positive	0 (0.0%)	54 (100.0%)	54 (15.3%)	298 (84.7%)	0.002	NA
Luminal B Her-2 Negative	23 (42.6%)	31 (57.4%)	104 (29.5%)	248 (70.5%)	0.054	1.769 (0.985–3.179)
Her-2 overexpression	1 (1.9%)	53 (98.1%)	34 (9.7%)	318 (90.3%)	1.000	0.176 (0.024–1.317)
TNBC	23 (42.6%)	31 (57.4%)	67 (19.0%)	285 (81.0%)	<0.001	3.156 (1.730–5.759)

**Notes.**

BRCA (+) BRCA mutation group BRCA (-) non-BRCA mutation group TNBC Triple negative breast cancer OR Odds ratio CI Confidence interval

**Table 5:** Comparative analysis of intergroup immunohistochemical indicators.

Characteristics	Grouping	Mann–Whitney			Negative rate (ER, PR, Her-2) / High expression ratio (Ki-67)	*P*-value
		Median (P25,P75)	Z	*P*		
ER	BRCA(+) BRCA(-)	30% (0%–80%) 80% (0%–90%)	−2.594	0.009	48.1% 32.7%	0.032
PR	BRCA(+) BRCA(-)	0% (0%–60%) 30% (0%–80%)	−3.469	<0.001	51.9% 33.5%	0.014
Her-2	BRCA(+) BRCA(-)	NA	NA	NA	98.1% 74.7%	<0.001
Ki–67	BRCA(+) BRCA(-)	40% (24%–60%) 25% (10%–40%)	−3.703	<0.001	83.3% 66.2%	0.012

**Notes.**

BRCA (+) BRCA mutation group BRCA (-) non-BRCA mutation group ER estrogen receptor PR progesterone receptor Her-2 Human epidermal growth factor receptor 2

NGS is an advanced BRCA gene mutation detection technology that can identify pathogenic variants, likely pathogenic variants, and variants of uncertain significance (Cao et al., 2019; Richards et al., 2008). Pathogenic variants and suspected pathogenic variants are usually analyzed together (Park et al., 2022) because they are associated with a high risk of hereditary breast cancer and ovarian cancer (Mehta et al., 2018). Variants of uncertain significance lack sufficient evidence to determine their pathogenicity (Dines et al., 2020). This study uses NGS technology to detect BRCA genes, defining pathogenic variants and likely pathogenic variants as the BRCA mutation group, while defining variants of uncertain significance and samples without detected BRCA mutations as the BRCA non-mutation group. Based on the 2018 (Wolff et al., 2018) and 2020 (Allison et al., 2020) American Society of Clinical Oncology/College of American Pathologists (ASCO/CAP) guidelines and the 2021 guidelines from the Chinese Anti-Cancer Association (Chinese Anti-Cancer Association CoBCS, 2021), we have established the positive and negative determination criteria for ER, PR, and Her-2. Based on the above criteria, breast cancer is classified into several subtypes, such as Luminal A, Luminal B, Her-2 overexpression, and TNBC.

In clinical practice, patients with breast cancer who have BRCA gene mutations often exhibit clinical characteristics that differ from those of non-mutated breast cancer patients. For example, in terms of age at diagnosis, BRCA mutation-related breast cancer cases are usually diagnosed at a younger age. Our study found that the average age of patients with BRCA mutation breast cancer is below 40 years, while in contrast, the average age of non-BRCA mutation cases is above 40 years. Breast cancer in patients with BRCA gene mutations typically presents with more specific pathological features, including high grade, invasive growth patterns, and a higher rate of lymphovascular invasion (Gul et al., 2024). Our study found that compared to the BRCA non-mutated group, tumors with BRCA mutations are more likely to be less than or equal to two centimeters, with significant statistical differences. This may be related to the relationship between BRCA genes and hereditary breast cancer. When a family member is diagnosed with breast cancer and BRCA mutation testing is positive, doctors usually recommend preventive measures, such as genetic testing for relatives and regular early screening (Dibble et al., 2022; Lei et al., 2022). This approach greatly increases the likelihood of early disease detection, allowing for timely treatment. From a histological perspective, cancer cells in patients with BRCA mutations are usually more irregular under the microscope. Histology is typically classified into grade I (well-differentiated), grade II (moderately differentiated), or grade III (poorly differentiated), with most tumors associated with BRCA mutations being grade III, indicating that BRCA mutation breast cancer has very unique characteristics (Daly et al., 2024; Fines, McCarthy & Buckley, 2025). Tumor cells often have larger nuclei and more prominent nucleoli, with more frequent cell division, indicating a higher nuclear grade. This suggests that breast cancer with BRCA mutations is more abnormal and more aggressive (El Hejjioui et al., 2023). Our research found significant differences in clinical and biological characteristics of BRCA-related breast cancer, including tumor size and histological grading. These characteristics not only affect treatment choices but may also influence survival outcomes (Pallonen et al., 2022). Observing tumor size and histological grade in BRCA mutation carriers can help doctors develop more precise treatment plans and better predict outcomes. Although many studies have explored the impact of BRCA mutations on breast cancer, there is still a lack of in-depth research to analyze the specific relationship between tumor size, histology, and prognosis (Li et al., 2024).

Different subtypes of breast cancer have distinct clinical manifestations and treatment responses. For example, Luminal A tumors generally have a better prognosis, while TNBC often performs poorly and does not respond to hormone therapy or HER2-targeted therapy (Caramelo et al., 2022; Nandi & Sharma, 2024; Silva & Mesquita, 2022). International studies indicate that breast cancer patients with BRCA gene mutations often present as TNBC (Suba, 2024). This study found that compared to patients without BRCA gene mutations, those with BRCA gene mutations are more likely to have TNBC, consistent with previous research. It is worth noting that when comparing the expression levels of immunohistochemical markers between the BRCA gene mutation group and the non-mutated BRCA gene group, it was found that the expression levels of ER and PR in the BRCA gene mutation group were lower, while the expression level of Ki-67 was higher. Considering the potential regional differences in BRCA gene mutation breast cancer, we searched the China National Knowledge Infrastructure (CNKI) database for relevant literature that conducted germline testing using the same testing methods. Chen’s (2007) study indicated that in northern regions, breast cancer patients with BRCA gene mutations tend to have a tumor pathological grade of III. Bao et al. (2024) and Fang (2018)’s research showed that in the central coastal region, the proportion of TNBC in the BRCA1 mutation group was higher than that in the non-mutation group. Additionally, two other studies in China showed that the proportion of TNBC among BRCA gene mutation carriers was higher than that in the non-mutation group, which is consistent with the findings of this study (Chen, 2019; Chen et al., 2022). BRCA gene mutations can affect the molecular subtypes of breast cancer, and these molecular subtypes help guide treatment choices and develop personalized treatment plans.

## Limitations

The limitations of our study mainly include the inherent limitations brought by retrospective analysis, which may lead to data bias. The group of BRCA mutation carriers only has 54 cases, which is relatively small compared to other groups, potentially undermining the reliability of the study results. The samples come from a single center and lack multi-center data, which limits the general applicability of the results and affects the accuracy of the analysis. In next-generation sequencing technology, the PCR amplification process may lead to an increased false positive rate (Xu et al., 2014). In addition, this technology has sequencing bias and cannot effectively detect structural variations of the BRCA gene and variations in regulatory regions (Wallace, 2016). Therefore, this study is exploratory.

## Conclusions

This study found that among breast cancer patients in Shenzhen, China, tumors carrying BRCA mutations were smaller in volume but had higher histological grades, and the molecular subtypes were more inclined towards triple-negative breast cancer. The rates of ER negativity, PR negativity, HER2 negativity, and high expression of Ki-67 in patients with BRCA mutations were significantly higher than those in patients without BRCA mutations. Therefore, our research deepens the understanding of the clinical characteristics of BRCA mutation-positive patients in the Shenzhen population.

##  Supplemental Information

10.7717/peerj.20813/supp-1 Supplemental Information 1 Raw data

10.7717/peerj.20813/supp-2 Supplemental Information 2 Comparison of germline mutations and somatic mutations in the BRCA gene

## Additional Information and Declarations

### Data Availability

The following information was supplied regarding data availability: The data is available in the Supplemental File.
