# Supplementary material for: Pathological features of BRCA-mutated breast cancer in Shenzhen, China: a single-center study
Source: PeerJ. 2026 Mar 3;14:e20813. doi: 10.7717/peerj.20813 (PMC12965168; doi:10.7717/peerj.20813)
Supplement: Supplemental Information 2 [file peerj-14-20813-s002.docx]

**Supplement table 2 :**Comparison of germline mutations and somatic mutations in the BRCA gene

| **Feature** | Germline mutation | Somatic mutation |
| --- | --- | --- |
| **Origin** | Germ cells | Tumor cells |
| **Detection tissue** | Blood | Tumor tissue |
| **Hereditary** | Heritable | Non-heritable |
| **Median age of illness** | BRCA1: 41.5 years old; BRCA2: 49.5 years old | 65 years old |
| **Proportion of occurrence** | 2/3 | 1/3 |
| **Similarities** | The sensitivity of PARP inhibitors and platinum drugs | |
|  | Display characteristics of homologous recombination deficiency | |
|  | Tumor phenotypic similarity | |
